# Decision regret and long-term weight evolution following laparoscopic sleeve gastrectomy as bridge to kidney transplantation

**DOI:** 10.3389/frtra.2025.1627504

**Published:** 2025-10-01

**Authors:** Xin Yu Yang, Pamela Brazeau-Porrello, Roy Hajjar, David Badrudin, Radu Pescarus, Gabriel Chan

**Affiliations:** ^1^ Division of General Surgery, Faculty of Medicine, Université de Montréal, Montréal, QC, Canada; ^2^ Service of Hepato-Pancreato-Biliary & Transplant Surgery, Hôpital Maisonneuve-Rosemont, Montréal, QC, Canada; ^3^ Service of Bariatric Surgery, Hôpital du Sacré-Cœur-de-Montréal, Montréal, QC, Canada

**Keywords:** laparoscopic sleeve gastrectomy, bariatric surgery, obesity, end-stage renal disease, kidney transplant, decision regret

## Abstract

**Introduction:**

Laparoscopic sleeve gastrectomy (LSG) is effective for rapid weight loss in kidney transplant (KT) candidates. This study aims to evaluate satisfaction or regret with the decision to undergo LSG in preparation for KT and the long-term durability of this approach to weight loss.

**Methods:**

From 2012 to 2019, all patients who underwent LSG prior to waitlisting for KT were included. The Decision Regret Scale (DRS) was assessed regarding the decision to undergo LSG before KT. The long-term weight evolution was also collected.

**Findings:**

Forty-six subjects completed the DRS survey at a median follow-up of 8 years post-LSG: 67% reported absolutely no regret, 22% mild regret, and 11% moderate to strong regret. Successful surgical weight loss was achieved in 36 patients and was significantly associated with lower levels of regret (*p* = 0.005). Body mass index reductions after LSG were highly significant compared to baseline values at all time points over 10 years (*p* = 0.0001) and remained significantly lower for up to 7 years post-KT. Thirty-two patients received KT, yet this had no significant association with decision regret.

**Conclusion:**

Laparoscopic sleeve gastrectomy as a pre-transplantation weight loss strategy is associated with very low levels of regret, regardless of the KT status. LSG has demonstrated long-term, durable weight loss.

## 1 Introduction

Laparoscopic sleeve gastrectomy (LSG) has been shown to be safe and effective for rapid weight loss in obese kidney transplant (KT) candidates (1–3). In addition to improvements in the metabolic syndrome, there is some evidence for the benefits of reduced delayed graft function, fewer graft losses, and improved survival (1, 4).

The peri-operative risks associated with LSG include staple line leak, sepsis and anesthesia-related complications. Long-term complications include gastroesophageal reflux, micronutrient deficiencies, dumping syndrome and gastric stenosis (1). Furthermore, weight loss is not assured, nor is the subsequent kidney transplantation. Recurrent obesity typically emerges between two and five years after LSG and may be further exacerbated following KT. Post-transplant weight gain can be driven by increased appetite due to improved overall health, lifting of the dietary restrictions previously required during dialysis and the side effects of immunosuppressive medications (5, 6). Regardless of the etiology, complications of bariatric surgery, failed weight loss, recurrent obesity or unattained transplantation, could each potentially lead to frustration and regret (7) about the decision to pursue bariatric surgery in preparation for kidney transplantation.

Bariatric surgery outcomes focus primarily on metrics like excess body weight loss or body mass index (BMI). However, these numbers may not fully capture the changes in quality of life nor, a patient's satisfaction or regret regarding this major clinical decision to proceed with bariatric surgery. The long-term evolution of BMI and the impact of the subsequent KT have also been less well documented. These objectives will hopefully provide guidance to transplant teams in assisting patients in the decision-making process during the preparation for transplantation.

## 2 Materials and methods

This was a clinical study aimed to evaluate decision regret among KT candidates undergoing bariatric surgery. The primary outcome was the Decision Regret Scale (DRS) regarding the previous clinical decision to undergo LSG to access the waiting list for KT. The DRS was administered during routine clinical follow-up visits or by telephone interviews between May 2025 and July 2025, with written or verbal informed consent obtained prior to participation. Secondary outcomes include the long-term evolution of weight after LSG and after KT. This study was approved by the Research Ethics Board at the Centre de Recherche de l'Hôpital Maisonneuve-Rosemont (CER#: 2019-1663).

### 2.1 Population

Patients were identified through the institutional Kidney Transplant Database. The inclusion criteria were an age of 18 years or older, diagnosis of end-stage renal disease undergoing evaluation for transplantation, and a history of LSG, between 2012 and 2019, prior to KT wait listing. Patients who had a LSG followed by a KT were included in the LSG2KT group. Patients who had a LSG and did not receive a KT, at the time of data collection, were included in the LSG-only group. The institutional policy regarding obesity uses a strict limit of a BMI less than 36.0 kg/m^2^ to be eligible for wait-listing. All patients with a BMI >35.0 kg/m^2^ or a BMI >30.0 kg/m^2^ with metabolic disorders were offered an opportunity for referral for bariatric surgery and LSG. The decision regarding a glucagon-like peptide-1 (GLP-1) receptor agonist for weight loss was made by the treating nephrologist or team.

### 2.2 Decision Regret Scale (DRS)

Patients' level of regret was assessed using the DRS, a validated tool known for its correlation with satisfaction in healthcare decisions and overall quality of life (8). The survey was administered via in-person interviews or standardized phone calls. Written or verbal informed consent was obtained from all participants before the interviews. Patients were asked to reflect on their decision to undergo LSG for KT eligibility using five items of the DRS scale:

1. It was the right decision.
2. I regret the choice that was made.
3. I would go for the same choice if I had to do it over again.
4. The choice did me a lot of harm.
5. The decision was wise.

Each item was rated on a 5-point Likert scale, ranging from 1 (strongly agree) to 5 (strongly disagree), with items 2 and 4 reverse-coded. The item score was calculated by converting to a 0–100 scale by subtracting 1 and multiplying by 25. A mean DRS score was then calculated for the five items. Decision regret levels were categorized into three groups based on the final score: no regret (score of 0), mild regret (score between 1 and 25) and moderate to strong regret (score between 26 and 100) (9).

### 2.3 Data collection

The data was collected from the medical chart included patient demographics, comorbidities, anthropometric measures, perioperative data, surgical complications, and graft function. BMIs were collected at 3, 6 and 12 months post-LSG and annually thereafter, including after KT. Metrics were calculated and reported in accordance with the guidelines of the American Society for Metabolic and Bariatric Surgery (10). Successful surgical weight loss (SSWL) was defined as achieving a BMI less than 36.0 kg/m^2^ or an excess weight loss greater than 50% within two years of LSG (3). Weight regain was characterized by a BMI increase of more than 5.0 kg/m^2^ from the lowest point achieved after LSG (3, 11). At our institution, the BMI cutoff for kidney transplant listing is <36.0 kg/m^2^. Patients who do not meet this criterion are followed every 3 months to reassess eligibility. No simultaneous laparoscopic sleeve gastrectomy and kidney transplantation procedures were performed at our center.

### 2.4 Statistical analysis

Statistical analysis was performed using GraphPad Prism 10 (version 10.3.1, GraphPad Software, LLC). Descriptive data were reported as median and range for continuous data and as proportions for categorical data. Intergroup comparisons for continuous data were performed using analysis of variance with *post hoc* Tukey's test after assessment of data normality and equality of variance assumptions. Categorical variables were compared using Fisher's exact test. Statistical significance was defined as a *P*-value of less than 0.05.

## 3 Results

**Figure 1:**
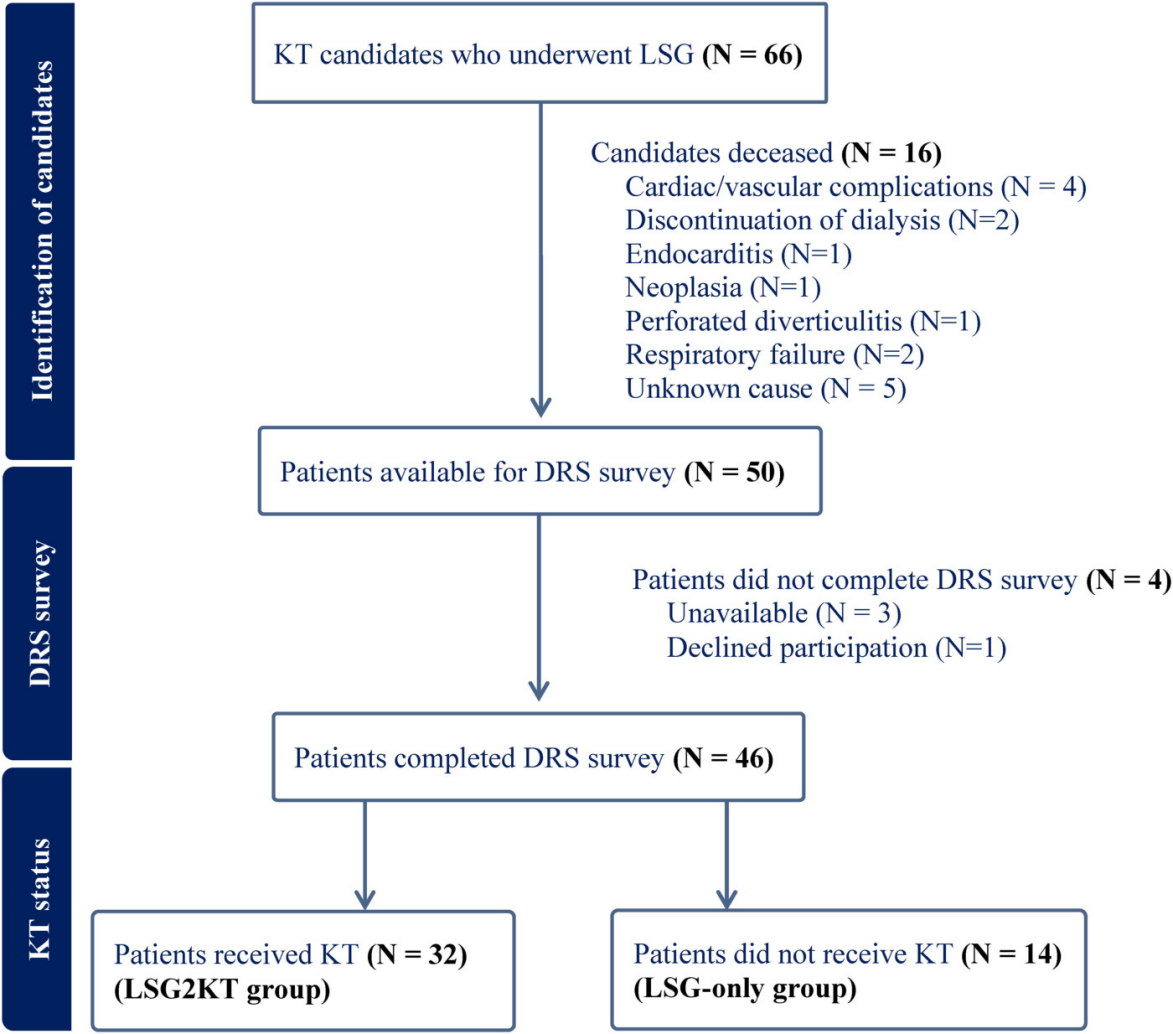
Flowchart illustrating the inclusion of kidney transplant (KT) candidates who underwent laparoscopic sleeve gastrectomy (LSG) in the study. A total of 66 patients underwent LSG. Sixteen were excluded due to death prior to survey administration. Of the remaining 50 patients, 4 did not complete the Decision Regret Scale (DRS) survey due to unavailability (*n* = 3) or declined participation (*n* = 1). A total of 46 patients completed the DRS survey and were subsequently divided into two groups based on transplant status: those who received KT (*n* = 32; LSG2KT group) and those who did not (*n* = 14; LSG-only group).

From 2012 to 2019, 66 KT candidates who underwent LSG were identified. Sixteen patients have died since, leaving fifty available to participate in the study. Four patients declined to participate in the DRS survey and interview. In total, forty-six patients completed the survey. A flowchart of the study participants is provided in Figure 1. The study cohort consisted of 30 (65%) males with a median age of 56 years at the time of the study survey. The median baseline BMI prior to LSG was 45.0 kg/m^2^. Most patients (*n*=43, 94%) were on dialysis at the time of LSG. Comorbidities at the time of LSG included: hypertension (*n*=24, 52%), dyslipidemia (*n*=17, 37%), diabetes mellitus (*n*=15, 33%), coronary artery disease (*n*=5, 11%) and sleep apnea (*n*=20, 44%). The patients' baseline demographic and clinical characteristics are depicted in Table 1. The demographic and anthropometric characteristics were stratified according to the level of DRS, and no significant differences were noted (Table 1).

**Table 1:** Baseline demographic and clinical characteristics by decision regret scale.

Characteristics		Total	No regret (DRS: 0)	Mild regret (DRS: 1–25)	Moderate to strong regret (DRS: 26–100)	*p*-value
*N*		46 (100%)	31 (67%)	10 (21%)	5 (10%)	
Age (years)^a^, median (range)		56 (33–80)	56 (39–80)	52.5 (43–64)	59 (33–61)	0.1678
Sex, *N* (%)	Female	16 (35%)	10 (32%)	5 (50%)	1 (20%)	0.5645
	Male	30 (65%)	21 (68%)	5 (50%)	4 (80%)	
Dialysis, *N* (%)		43 (94%)	30 (97%)	9 (90%)	4 (80%)	0.2444
Comorbidities, *N* (%)	Hypertension	24 (52%)	15 (48%)	4 (40%)	5 (100%)	0.0740
	Dyslipidemia	17 (37%)	10 (32%)	4 (40%)	3 (60%)	0.4633
	Diabetes mellitus	15 (33%)	11 (35%)	1 (10%)	3 (60%)	0.1238
	Coronary artery disease	5 (11%)	3 (10%)	0	2 (40%)	0.2034
	Sleep apnea	20 (44%)	15 (48%)	2 (20%)	3 (60%)	0.2487
Initial BMI at LSG (kg/m^2^), median (range)		45.0 (40.7–48.4)	43.3 (40.8–48.4)	46.3 (41.7–48.5)	43.7 (41.0–47.8)	0.8873

^a^ At the time of the questionnaire; LSG, laparoscopic sleeve gastrectomy; BMI, body mass index.

**Figure 2:**
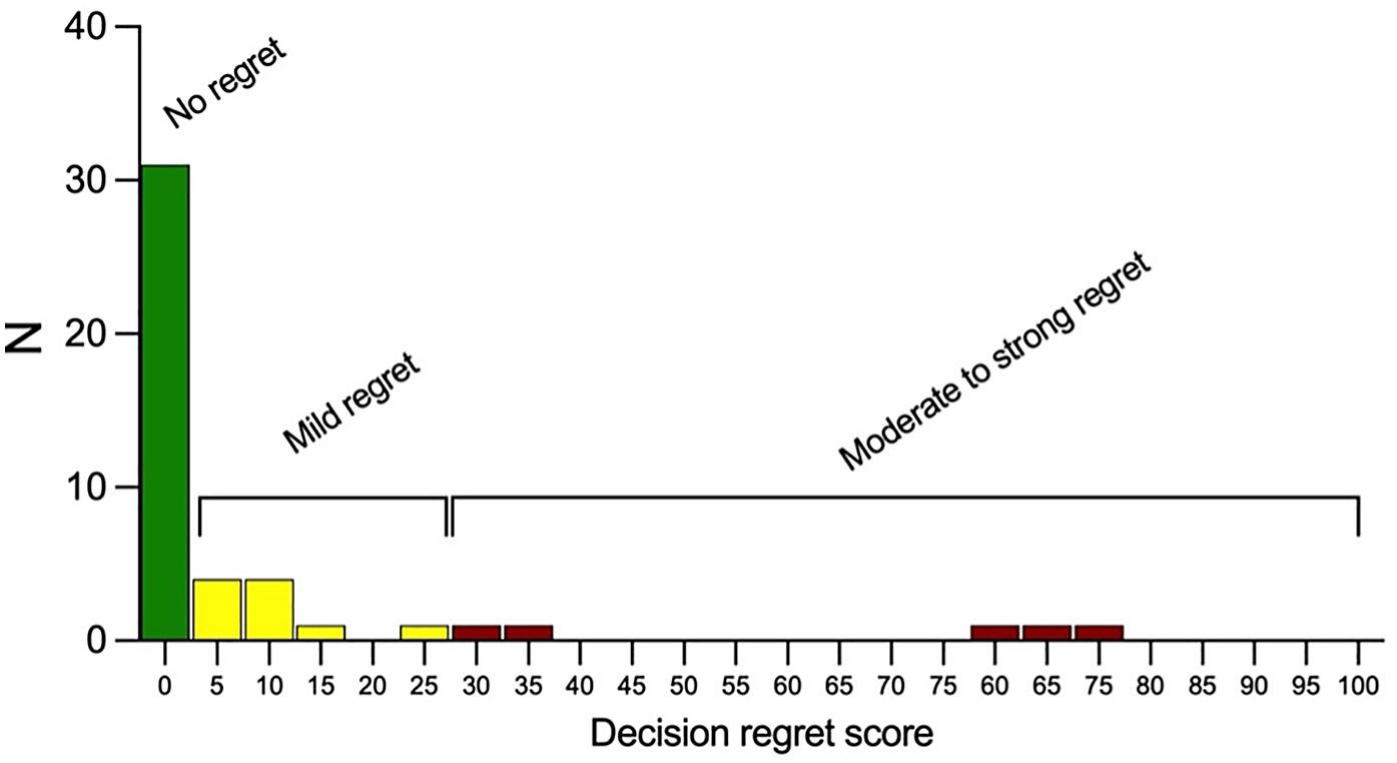
Distribution of decision regret scores among study participants. Scores range from 0 to 100, with categories of “No regret” (0), “Mild regret” (1–25), and “Moderate to strong regret” (26–100). The majority of patients reported no or mild regret.

The mean overall DRS score was 0 (range: 0–75). The score was reported at a median of 8 years after LSG. The distribution of scores is depicted in Figure 2. Thirty-one patients (67%) reported absolutely no regret with a score of 0. Mild regret (score 1–25) was reported by 10 patients (22%), while 5 individuals (11%) expressed moderate to strong regret (scores 26–100).

SSWL was achieved in 36 patients (78%), of whom, 27 (75%) reported no regret, 4 (11%) expressed mild regret, and 5 (14%) reported moderate to strong regret. A lower level of regret was significantly associated with the SSWL (*p*-value = 0.005). Surgical weight loss after LSG is summarized in Table 2. During our interviews, patients specifically mentioned changes in their relationship with food, excessive weight loss and psychological distress from altered interpersonal relationships as reasons for their regret. These items are listed in (Supplementary Material S1).

**Table 2:** Outcomes after laparoscopic sleeve gastrectomy by decision regret scale.

Outcomes		Total	No regret (DRS: 0)	Mild regret (DRS: 1–25)	Moderate to strong regret (DRS: 26–100)	*p*-value
Follow-up after LSG (years), median (range)		8 (5–10)	7 (2–23)	9 (4–13)	8 (5–10)	0.7534
Successful surgical weight loss, *N* (%)	Yes	36	27 (87%)	4 (40%)	5 (100%)	0.005
	No	10	4 (13%)	6 (60%)	0	
Transplanted, *N* (%)	Yes	32	22 (71%)	6 (60%)	4 (80%)	0.7882
	No	14	9 (29%)	4 (40%)	1 (10%)	
Weight regain after LSG, *N* (%)	Yes	11	7 (23%)	3 (30%)	1 (20%)	0.8670
	No	35	24 (77%)	7 (70%)	4 (80%)	

Among the study participants, 32 individuals (70%) received a kidney transplant following their LSG (LSG2KT group), while 14 did not (LSG-only group). Three patients received a graft (9%) from a living donor. Overall, there were no statistically significant differences in graft outcomes between patients with no regret and those with mild or moderate-to-strong regret. Specifically, the occurrence of wound, urinary, vascular, or any complications, as well as readmissions, re-interventions, or re-operations within 90 days, did not differ significantly between groups (all *p*-values >0.22). Regarding graft-specific outcomes, 13 patients (41%) experienced delayed graft function, and no cases of primary non-function were observed. Biopsy-proven acute rejection occurred in 4 patients (13%) within the first year post-KT, with no association between these outcomes and decision regret. Two patients experienced graft loss and returned to dialysis: one due to rejection two years post-transplant and one due to ureteric stenosis with sepsis four years post-transplant; both reported no regret regarding their decision to undergo LSG prior to KT.

Within the LSG2KT group, 22 patients (69%) reported absolutely no regret, 6 expressed mild regret, and 4 experienced moderate to strong regret. In comparison, among the LSG-only group (n=14), 9 patients (64%) reported no regret, 4 (29%) expressed mild regret, and 1 (7%) experienced moderate to strong regret. There was no significant difference in regret based on achieving KT (*p*-value = 0.7882). In the LSG-only group, two patients were removed from the transplant list due to worsening comorbidities or cancer, and five failed to be listed, due to non-compliance (*n* = 1) and failed surgical weight loss (*n* = 4). Seven patients are currently still on the waiting list for KT.

**Figure 3:**
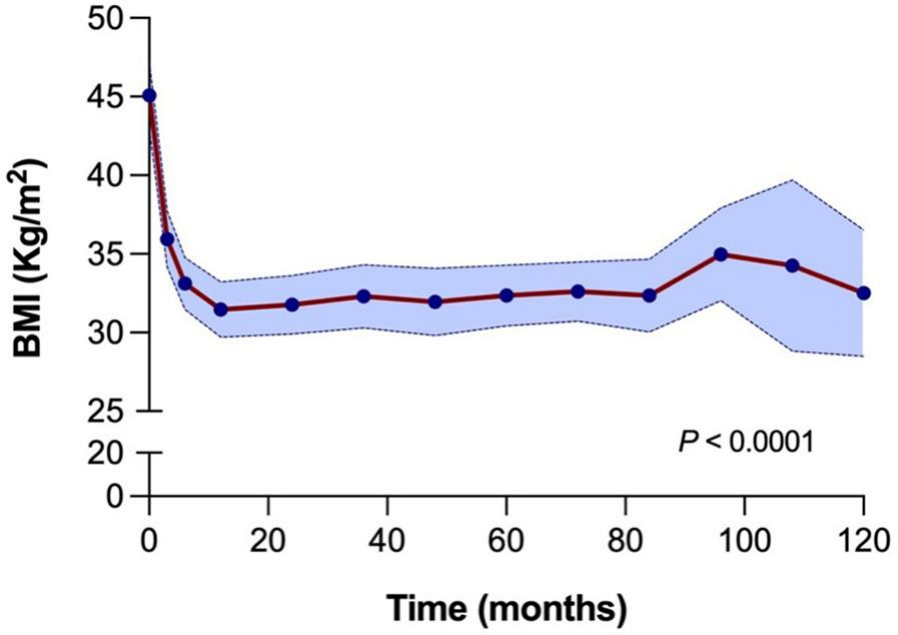
BMI evolution over 10 years (120 months) following laparoscopic sleeve gastrectomy. A significant reduction in BMI is observed shortly after surgery, with sustained lower BMI over time. The blue shaded area indicates the 95% confidence interval. Statistical comparisons are made between all-time points and baseline (time 0), with a *p*-value < 0.0001.

The long-term evolution of BMI following LSG demonstrated sustained and significant weight loss, as illustrated in Figure 3. The median BMI decreased from 45.0 kg/m^2^ (interquartile range 41.3–48.4) at the time of surgery to 35.3 kg/m^2^ (33.0–37.4) at three months and 32.5 kg/m^2^ (30.7–35.2) at six months postoperatively. A gradual stabilization of BMI was observed between one to seven years postoperatively, with BMI values ranging between 31.2 kg/m^2^ and 32.7 kg/m^2^. Between eight and nine years, there was a slight increase in median BMI at 36.5 kg/m^2^ and 36.0 kg/m^2^. After ten years, the median BMI was 32.1 kg/m^2^ (30.8–34.1), highlighting the durability of weight loss over the long term (Supplementary Material S2). The decrease in BMI at all time points post-LSG was significant compared to baseline values, even up to ten years postoperatively (*p*-value < 0.0001). This highlights the durability of surgical weight loss of LSG in renal transplant candidate and recipient populations. There were no statistically significant differences in long-term weight evolution following LSG between the regret groups at any time point up to ten years after LSG (Supplementary Material S3). Weight regain following LSG was observed in 11 patients (24%) at a median time of three years. Two patients with weight regain have received GLP-1 receptor agonists. Weight regain did not show a significant association with decision regret.

**Figure 4:**
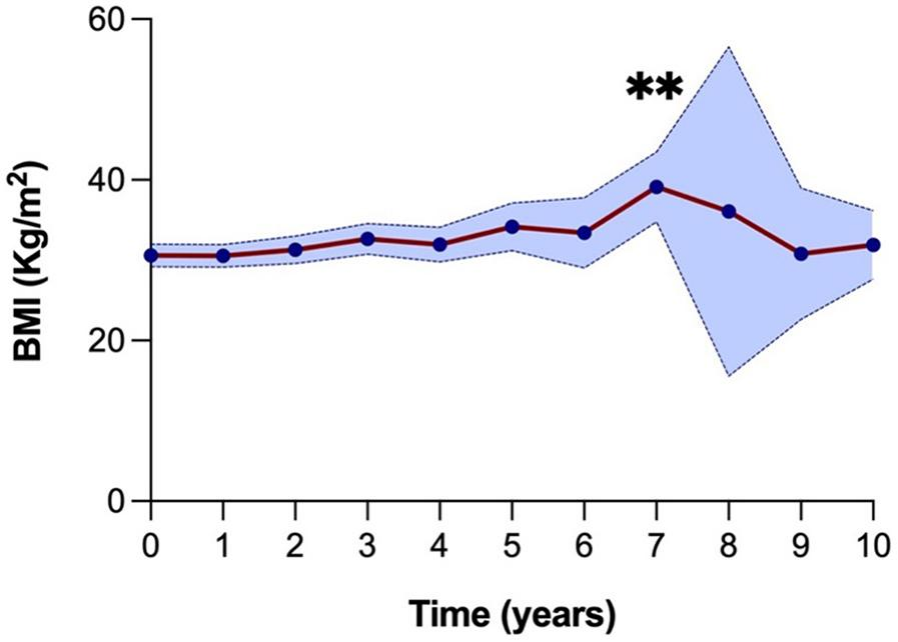
BMI evolution over 10 years (120 months) following kidney transplantation. A significant increase in BMI was observed at year 7 compared to baseline (**P = 0.01). The blue shaded area represents the 95% confidence interval.

The median BMI at the time of KT was 31.8 kg/m^2^ (27.7–33.5) (Figure 4). For the first four years post-KT, the median BMI remained relatively stable around 31 kg/m^2^. This was followed by a gradual rise, reaching 33.5 kg/m^2^ (29.9–38.6) at five years and 32.9 kg/m^2^ (30.1–36.7) at six years. There was a notable peak at year seven, with a median BMI of 39 kg/m^2^ (35.6–41.9), marking a statistically significant difference compared to the baseline. Thereafter, median BMI values tended to be lower and reached 31.7 kg/m^2^ (29.9–34.0) at ten years post-KT (Supplementary Material S4).

Weight regain after transplantation occurred in 10 (31%) recipients, but the rate of weight regain was not significantly associated with levels of decision regret. Interestingly, a lower BMI within the first three years post-KT was significantly associated with a higher level of regret. The median BMI at one year of patients with no regret was 31.3 kg/m^2^, with mild regret, 27.4 kg/m^2^, and moderate to strong regret, 23.4 kg/m^2^ (*p*-value = 0.009). At two years post-KT, median BMIs were 31.7 kg/m^2^ in the no regret group, 28.8 kg/m^2^ in the mild regret group and 24.9 kg/m^2^ in the moderate to strong regret group (*p*-value = 0.0399). At three years post-KT, median BMIs were 35.8 kg/m^2^ in the no-regret group, 30.2 kg/m^2^ in the mild regret group and 25.9 kg/m^2^ in the moderate to strong regret group (*p*-value = 0.0475). Beyond three years, BMI values stabilized, and differences between regret groups were no longer statistically significant (Supplementary Material S5).

## 4 Discussion

The rapid and dramatic weight loss provided by LSG for patients in preparation for KT is well-established (12, 13). To date, few studies have reported the long-term outcomes of this approach. In this present cohort of KT candidates, the majority achieved SSWL after LSG (78%), with a median change in BMI of 12.9 kg/m^2^ at 10 years, and almost as many (70%) received a subsequent KT. Furthermore, the rapid surgical weight loss was sustained for up to ten years post-LSG and up to six years after the subsequent kidney transplantation. A cohort study by Zaminpeyma et al. found that the median change in BMI (−10.5 kg/m^2^) was maintained at five years post-transplantation in recipients with a previous LSG (14). Cohen et al. documented a slight increase in median BMI post-transplantation at five years from 32 kg/m^2^ to 36 kg/m^2^ in a heterogeneous cohort of 43 recipients with a history of various types of bariatric surgery (15). This study adds promise for a durable surgical weight loss for KT candidates, which is maintained even after a subsequent KT with the concomitant immunosuppressive regimens. On the other hand, it should be noted that there is still a minority who may never reach transplantation for a variety of reasons, including failed surgical weight loss. It is also conceivable that transplantation rates could be improved if more candidates had had a living donor.

The survey response rate was high, with 92% eligible participants consenting to answer the Decision Regret Scale, over a median follow-up period post-LSG of 8 years. The pattern favoured a high degree of satisfaction, with 67% of respondents reporting absolutely no regret and 22% having only mild regret. A minority (11%) had moderate to strong regret. These DRS results are similar to those of Dijkhorst et al., who reported results of post-LSG in a general population: 50% reported no regret, 35% mild levels, and 15% moderate to strong regret (9). The vast majority of kidney transplant candidates (89%) in this study harboured little or no regret. This low level of regret represented an important clinical outcome to support of the LSG option for KT candidates.

Successful surgical weight loss was associated with significantly lower regret. This aligns with findings from other studies, which have highlighted the positive correlation between successful weight loss and lower levels of decision regret (7, 16). Interestingly, there was no statistically significant difference in the DRS between the LSG2KT and LSG-only subgroups. In other words, achieving KT or not, did not have a measurable impact on regret of the previous decision to undergo LSG to prepare for KT. This finding may highlight the broader benefits of SSWL, which can improve overall health and quality of life (17, 18). Having chosen to undergo bariatric surgery in pursuit of the goal of KT, may give patients a sense of having done everything possible to achieve their objectives. Despite the failed weight loss, they had no regrets.

The DRS findings revealed another unexpected nuance: the minority who reported moderate to strong regret (*n* = 5) had achieved weight loss at the higher range, perhaps excessively so. None of the patients in the unsuccessful weight loss group had moderate to strong regret. This pattern was also observed following KT. From the first year to the third year, the patients with moderate to strong regret had significantly lower BMI than those with mild regret and those with no regret. Substantial weight loss can result in a negative body image perception, which may pose a psychological challenge for some individuals (19–21), making it difficult to accept their new body (22). The specific concerns expressed by patients with moderate to strong regret followed two main themes: difficult dietary changes and on-going mood disturbances. Patients had a difficult time accepting the limitations on diet due to the restrictive surgery, the loss of enjoyment from eating and the complication of dumping syndrome. All these had a negative impact on their relationship with food. Mood changes included feelings of abandonment or lack of support, depression (attributed to weight loss after LSG) and anxiety about eating disorders. Weight regain was also cited regarding regret. Although reasonably uncommon, these findings of significant regret underscore the importance of individualized patient counselling pre- and post-bariatric surgery. Managing expectations, addressing body image concerns and mood disorders, and providing tailored nutritional and psychological support could mitigate regret and improve long-term patient satisfaction (23).

This study is limited by the size of the study cohort, preventing further inter-comparisons to identify predictors of regret. Future multicentre studies with surveys of wider populations will help confirm the overall generalizability of these results. A selection or participation bias favouring satisfaction is certainly possible. Future studies will also have to take into consideration GLP-1 analogues and compare their long-term efficacy for weight loss versus LSG or other bariatric operations.

## 5 Conclusion

This study has demonstrated that the rapid weight loss from laparoscopic sleeve gastrectomy for KT candidates is also durable and maintained after a subsequent kidney transplant. Moreover, the clinical decision to choose this path of LSG prior to KT is associated with low overall regret, surprisingly, regardless of receiving a kidney transplant or not. Counselling regarding the psychological adaptations after surgical weight loss and education as to the expected changes to dietary habits could mitigate the small but important areas of regret.

## Data Availability

The raw data supporting the conclusions of this article will be made available by the authors, without undue reservation.
