# Supplementary material for: Decision regret and long-term weight evolution following laparoscopic sleeve gastrectomy as bridge to kidney transplantation
Source: Front Transplant. 2025 Oct 1;4:1627504. doi: 10.3389/frtra.2025.1627504 (PMC12521130; doi:10.3389/frtra.2025.1627504)
Supplement: Supplementary file 1 [file Table1.docx]

**Supplementary Material 1.** Patient-reported items related to decision regret

| “[I] regained the lost weight after bariatric surgery during a depressive episode.” |
| --- |
| “[I] know a friend who had bariatric surgery, developed an eating disorder and post-surgery depression, and committed suicide.” |
| “[…] lack of bariatric and psychological support […]” |
| “Limitations related to diet after bariatric surgery.” |
| “[I] have dumping syndrome symptoms […] not able to enjoy food as much as before.” |
| “[I] would have liked to try other weight loss alternatives first.” |
