# Supplementary material for: Decision regret and long-term weight evolution following laparoscopic sleeve gastrectomy as bridge to kidney transplantation
Source: Front Transplant. 2025 Oct 1;4:1627504. doi: 10.3389/frtra.2025.1627504 (PMC12521130; doi:10.3389/frtra.2025.1627504)
Supplement: Supplementary file 2 [file Table2.docx]

**Supplementary Material 2.** Body mass index evolution after laparoscopic sleeve gastrectomy

| **Time after LSG (months)** | **BMI (Kg/m²), median (range)** |
| --- | --- |
| At LSG (N=46) | 43.0 (31.0-65.9) |
| 3 (N=30) | 35.4 (26.9-48.3) |
| 6 (N=25) | 32.5 (26.9-46.4) |
| 12 (N=43) | 31.4 (22.4-46.7) |
| 24 (N=36) | 31.5 (21.7-46.7) |
| 36 (N=33) | 32.3 (21.8-43.9) |
| 48 (N=29) | 32.0 (21.0-44.2) |
| 60 (N=33) | 31.9 (21.9-43.3) |
| 72 (N=27) | 32.7 (21.1-39.9) |
| 84 (N=18) | 31.5 (23.6-43.0) |
| 96 (N=13) | 36.5 (28.7-42.9) |
| 108 (N=6) | 36.0 (26.9-38.6) |
| 120 (N=10) | 32.1 (20.4-40.9) |
