# Supplementary material for: Decision regret and long-term weight evolution following laparoscopic sleeve gastrectomy as bridge to kidney transplantation
Source: Front Transplant. 2025 Oct 1;4:1627504. doi: 10.3389/frtra.2025.1627504 (PMC12521130; doi:10.3389/frtra.2025.1627504)
Supplement: Supplementary file 3 [file Table3.docx]

**Supplementary Material 3.** Body mass index evolution after kidney transplantation

| **Time after KT (months)** | **BMI (Kg/m²), median (range)** |
| --- | --- |
| At KT (N=37) | 31.8 (20.7-37.0) |
| 12 (N=34) | 31.2 (21.7-39.7) |
| 24 (N=31) | 31.2 (21.8-39.6) |
| 36 (N=26) | 32.5 (22.3-39.6) |
| 48 (N=18) | 31.1 (26.0-39.0) |
| 60 (N=13) | 33.5 (26.8-40.6) |
| 72 (N=8) | 33.1 (23.6-39.5) |
| 84 (N=5) | 40.5 (34.1-43.0) |
| 96 (N=3) | 38.5 (26.9-42.9) |
| 108 (N=5) | 32.0 (20.4-38.6) |
| 120 (N=4) | 31.7 (29.0-35.3) |
