# Supplementary material for: Decision regret and long-term weight evolution following laparoscopic sleeve gastrectomy as bridge to kidney transplantation
Source: Front Transplant. 2025 Oct 1;4:1627504. doi: 10.3389/frtra.2025.1627504 (PMC12521130; doi:10.3389/frtra.2025.1627504)
Supplement: Supplementary file 4 [file Table4.docx]

**Supplementary Material 4.** Body mass index evolution after kidney transplantation

| **Time after KT (months)** | **BMI (Kg/m²), median (range)** |
| --- | --- |
| At KT (N=32) | 31.8 (27.7–33.5) |
| 12 (N=29) | 30.7 (28.7–32.0) |
| 24 (N=27) | 30.8 (28.9–33.4) |
| 36 (N=26) | 31.6 (29.4–37.0) |
| 48 (N=18) | 31.1 (29.0–35.8) |
| 60 (N=13) | 33.5 (29.9–38.6) |
| 72 (N=8) | 32.9 (30.1–36.7) |
| 84 (N=5) | 39.0 (35.6–41.9) |
| 96 (N=3) | 35.4 (26.9–42.8) |
| 108 (N=5) | 32.0 (30.8–32.2) |
| 120 (N=4) | 31.7 (29.9–34.0) |
