# Supplementary material for: Decision regret and long-term weight evolution following laparoscopic sleeve gastrectomy as bridge to kidney transplantation
Source: Front Transplant. 2025 Oct 1;4:1627504. doi: 10.3389/frtra.2025.1627504 (PMC12521130; doi:10.3389/frtra.2025.1627504)
Supplement: Supplementary file 5 [file Table5.docx]

**Supplementary Material 5.** Body mass index evolution after kidney transplantation by decision regret scale

| **Time after KT (months)** | **BMI (Kg/m²), median (range)** | | | |
| --- | --- | --- | --- | --- |
|  | **No regret** | **Mild regret** | **Moderate to strong regret** | **p-value** |
| At KT (N=32) | 32.2 (29.8-40.0) | 27.8 (23.7-32.0) | 28.1 (23.2-33.2) | 0.1526 |
| 12 (N=29) | 31.3 (30.0-33.2) | 27.4 (24.1-31.2) | 23.4 (21.7-32.1) | 0.0090 |
| 24 (N=27) | 31.7 (30.6-35.3) | 28.8 (26.6-33.5) | 24.9 (22.9-29.6) | 0.0399 |
| 36 (N=26) | 35.8 (31.2-38.2) | 30.2 (28.2-33.5) | 25.9 (22.3-29.6) | 0.0475 |
| 48 (N=18) | 32.3 (29.7-36.1) | 30.9 (28.2-35.1) | 27.6 (26.8-29.2) | 0.3405 |
| 60 (N=13) | 36.7 (32.2-39.9) | 33.1 (30.2-36.8) | 28.4 (26.8-30.0) | 0.1303 |
| 120 (N=4) | 31.7 (29.9-34.0) | NA | NA | NA |
